# Milk Polysialic Acid Levels Rapidly Decrease in Line with the N-Acetylneuraminic Acid Concentrations during Early Lactation in Dairy Cows

**DOI:** 10.3390/biology12010005

**Published:** 2022-12-20

**Authors:** Julia Hinterseher, Juliane Günther, Kristina Zlatina, Lisa Isernhagen, Torsten Viergutz, Elisa Wirthgen, Andreas Hoeflich, Andreas Vernunft, Sebastian Peter Galuska

**Affiliations:** 1 Department of Dermatology and Allergology, Philipps University Marburg, Baldingerstraße 1, 35043 Marburg, Germany; 2 Research Institute for Farm Animal Biology (FBN), Wilhelm-Stahl-Allee 2, 18196 Dummerstorf, Germany; 3 Department of Pediatrics, Rostock University Medical Center, 18057 Rostock, Germany

**Keywords:** polysialic acid, sialic acids, polysialyltransferases, milk, dairy cow, mastitis

## Abstract

**Simple Summary:**

In addition to their function as energy sources, monosaccharides are used to build complex structured oligo- and polysaccharides, which play numerous essential roles as functional biomolecules. Such bioactive sugars are also key components of milk, since they have positive impacts on intestinal development, the gut microbiome, and an effective immune system, along with the learning and memory ability of offspring. Moreover, milk oligo- and polysaccharides have anti-adhesive properties against pathogenic microorganisms and viruses and are, therefore, important for the health of the mammary gland and the offspring. One key monosaccharide of such oligo- and polysaccharides is the sialic acid N-acetylneuraminic acid (Neu5Ac). Since bovine milk is not only important for calf health but also, in the case of colostrum, as a functional food for humans, it is of particular interest, at which time of lactation the highest amounts of these bioactive molecules are found in bovine milk. Our results demonstrate that on the day of calving, the highest amounts of Neu5Ac and its polymers are present in bovine milk and, thus, the sialic acid-dependent benefits of bovine milk are also highest at this time.

**Abstract:**

Sialylated milk oligosaccharides and glycoconjugates have several positive effects on the mucosal barrier, the gut microbiome, and an effective immune system. For this reason, they are important biomolecules for mammary gland health and optimal development of offspring. In milk, the major sialic acid, N-acetylneuraminic acid (Neu5Ac), can be attached as monosialyl-residues or as polymers. To investigate the sialylation processes during lactation of German Holstein cows, we analyzed udder tissue in addition to milk at different time points of lactation. The analysis of the milk samples revealed that both the levels of Neu5Ac and its polymer, polysialic acid (polySia), rapidly decreased during the first three days of lactation, and a high interindividual variance was observed. In mature milk, however, the sialylation status remains relatively constant. The results indicate that mammary gland epithelial cells are one source for milk polySia, since immunohistochemistry of udder tissue exhibited strong polySia staining in these cells. Furthermore, both polysialyltransferases, ST8SiaII and ST8SiaIV, are expressed. Based on known functions of monosialyl residues and polySia, we discuss the potential impact of these biomolecules and the consequences of the heterogeneous sialylation status of milk in relation to udder health and offspring health.

## 1. Introduction

N-Acetylneuraminic acid (Neu5Ac) and other members of the sialic acid family are acidic monosaccharides. They are essential functional units of numerous glycoproteins and glycolipids, as well as freestanding oligosaccharides [1,2,3,4,5,6].

In milk, sialylated oligo- and polysaccharides play several different roles and are important for the optimal development of offspring. Examples are their positive impact on intestinal development, the mucosal barrier, the gut microbiome, and an effective immune system [5,7,8,9,10,11]. In addition, soluble sialylated glycans have anti-adhesive properties against pathogenic microorganisms and viruses [8,9]. Moreover, the development of the brain and the nervous system seems to be supported by milk sialic acids, resulting in increased learning and memory ability [7]. As Spichtig et al. mentioned, these numerous positive aspects are the reason why companies frequently label their products for infants with the sialic acid content [12].

Sialic acids are mostly present as monomers on glycoconjugates, but linear sialic acid chains with a degree of polymerization between two and more than 100 sialic acid units are also produced in vertebrates [13,14,15]. The elongation of oligo- and polysaccharides with monosialyl residues takes place in all cells of vertebrates. In contrast, the synthesis of polysialic acid (polySia) is commonly restricted to selected cell types. In vertebrates, two polysialyltransferases are known, ST8SiaII and ST8SiaIV [13]. Interestingly, both enzymes are frequently expressed in parallel in polySia-positive cells. Thus, both polysialyltransferases can polysialylate in parallel cellular glycoproteins in the Golgi-apparatus.

In mammals, only polySia chains consisting of Neu5Ac have been described so far and, interestingly, such polymers are also present in human and murine milk [16,17]. Several different roles of polySia have been described. As a part of the glycocalyx on the cell surface, polySia chains inhibit cell adhesion, which supports, for instance, the migration of cells [18,19,20]. This also applies to cancer metastasis, since polySia can increase the motility of cancer cells [21,22,23,24,25].

In addition, polySia binds growth factors, such as the brain-derived neurotrophic factor (BDNF), the basic fibroblast growth factor (bFGF), and the vascular endothelial growth factor (VEGF), and seems to modulate their activity [26,27,28]. Intriguingly, a protective effect for peptides against proteolytic cleavage has also been described when they are bound to polySia [27]. This might be important when polySia interacts with growth factors or antimicrobial peptides, such as lactoferricin [27,29,30,31]. Interestingly, polySia also binds and modulates the activity of lactoferrin, one of milk’s major bioactive proteins [17]. Moreover, human milk polySia was suggested as a significant source of exogenous sialic acids for offspring [16].

However, the polySia concentration of bovine milk during different phases of lactation is unknown. For this reason, we analyzed the milk of German Holstein cows at different time points of lactation, as well as udder tissue, to investigate the polysialylation in dairy cows.

## 2. Materials and Methods

### 2.1. Milk and Tissue Samples

The tissues and cells used for the expression analyses were obtained from healthy, first-lactating Holstein–Friesian dairy cows. The animals were slaughtered in the FBN’s own abattoir (EU license ES1635) in compliance with all necessary ethical and legal requirements. The preparation and cultivation of primary bovine mammary gland epithelial cells (MECs) were performed as previously described [32]. For the immunohistochemical experiments, formalin-fixed and paraffin-embedded mammary gland tissues were used, which were obtained from noninfected udder quarters in an animal trial, as previously described [33].

The milk samples were taken from five Holstein–Friesian dairy cows housed in the FBN’s experimental cattle facility. Four cows were on their second calf and one was on her fourth. Milk was collected at eight time points: during pregnancy (approximately d40, d135, and d220 after conception), after calving (colostrum d0, d1, and d2 postpartum), and around the 7th and 30th postpartum days of the next lactation. The colostrum on day 0 was collected shortly after the end of calving (first milk, 1–5 h). The other samples were collected manually before regular milking in the morning.

In contrast to the milk samples of Holstein–Friesian dairy cows, mature milk samples of all other mammals were not collected at defined time points. The mature milk samples were collected, at the earliest, 14 days after birth. Buffalo milk samples were kindly provided by Milchqualitätsdienst (MQD) Güstrow, Germany. Milk from dwarf goats was collected in Lelkendorf (Haustierpark Lelkendorf, Germany). Sheep milk samples were shared by the Schafsscheune Vietschow (Vietschow, Germany). Milk from Mecklenburger warm blood horses was collected at the FBN. Porcine milk was obtained from German landrace sows in the FBN’s experimental pig facility. Human milk samples were kindly provided by Clemens Kunz JLU Giessen (approved by the ethics office of the University of Giessen, School of Medicine (ID77/00)). All milk samples were stored at −20 °C in aliquots until use.

### 2.2. Quantification of Neu5Ac in Milk

Neu5Ac was quantified by reversed-phase (RP) HPLC, as described earlier [34,35]. For this, milk samples were initially diluted 1:100 in ddH_2_O and hydrolyzed with 0.2 M TFA (trifluoroacetic acid, Carl Roth GmbH + Co. KG, Karlsruhe, Germany) at 80 °C for 4 h. Subsequently, they were dried and labeled with DMB (4,5-methylene dioxybenzene; Dojindo, Kumamoto, Japan) in 80 μL DMB reaction buffer (9 mM sodium hydrosulfite, 0.5 M β-mercaptoethanol and 20 mM TFA) at 55 °C for 2 h. The reaction was stopped by the addition of 20 μL 0.2 M NaOH. As a standard, a sialic acid mixture containing KDN (ketodeoxynonulosonic acid, Sigma Aldrich, Taufkirchen, Germany), Neu5Gc (N-glycolylneuraminic acid, Sigma Aldrich, Taufkirchen, Germany), and Neu5Ac (Carbosynth, Compton, UK) was used and treated in the same way as the samples. The separation was performed on a Superspher ^®^ 100 RP-18 end-capped column (250 mm × 40 mm, Merck-Hitachi, Darmstadt, Germany) at 55 °C. The two eluents, methanol/acetonitrile/water/TFA (4:4:92:0.1; E1) and methanol/acetonitrile/water/TFA (45:45:10:0.1; E2), were used with a linear gradient from 0% to 5% E2 over 25 min at a flow rate of 0.25 mL/min. The subsequent gradient was 31 to 40 min 100% E2 and 41 to 55 min 0% E2 with the same flow rate. A fluorescence detector (372 nm for excitation and 456 nm for emission) detected the signals.

### 2.3. Western Blotting

PolySia was isolated from milk using inactivated endoneuraminidase (endoN), which was covalently coupled to tosylactivated Dynabeads^®^ M-280 (Life Technologies, Oslo, Norway), as described previously [17,36]. For negative control, aliquots of all samples were treated with active endoN (1 h at 37 °C) to degrade polySia and, thus, to abolish the binding of the primary antibody. The resulting samples were separated by SDS-PAGE (7% gel) using reducing conditions and subsequently transferred onto a PVDF membrane. Immunostaining against polySia was performed with the monoclonal antibody (mAb) 735 (1 µg/mL). The mAb 735 was provided by Martina Mühlenhoff (MHH, Hannover Germany). The mAb was produced in her laboratory. Horseradish peroxidase (HRP)-conjugated secondary antibodies (Dako, Hamburg, Germany) were applied for visualization of the bound primary mAb. The chemiluminescence signal was recorded with a ChemDoc MP Imaging system (Bio-Rad, Feldkirchen, Germany).

### 2.4. Expression Analysis

Total RNA was extracted from bovine udder tissue and cultured primary bovine MECs with the Direct-zol RNA MiniPrep Kit (Zymo Research, Freiburg, Germany). Subsequently, cDNA was prepared from 200 ng of each RNA using the iScript cDNA Synthesis Kit (Bio-Rad Laboratories, Munich, Germany). As positive controls for *ST8SIA2* and *ST8SIA4* primers, RNA from bovine brain and lung tissue were extracted in parallel. The abundance of *ST8SIA2* and *ST8SIA4* mRNA was determined using iQ SYBR Green Supermix and CFX96 Touch Real-Time PCR Detection System (both from Bio-Rad, Germany). The following primer pairs (sense and anti-sense, respectively) were used to measure *ST8SIA2* 5′-CCAGCTGTTGTTGACAGAAGTAAT-3′/5′-TCTCGGTCGAAGATGTAATGAATA-3′ and *ST8SIA4* 5′-TCTGGCATTCTGCTAGATAGTGAG-3′/5′-TGTCATTCAGCATGGAAAGTCTAT-3′ (TIB Molbiol, Berlin, Germany). The PCR cycle parameters were as follows: initial denaturation at 94 °C for 3 min, followed by 40 cycles at 94 °C for 10 sec, 60 °C for 30 sec, 70 °C for 45 sec, and post-extension at 70 °C for 7 min. The 197 bp (*ST8SIA2*) and 220 bp (*ST8SIA4*) PCR amplicons were visualized on 2% agarose gels. Sanger sequencing confirmed the specificity of the PCR fragments.

### 2.5. Immunohistochemistry

Paraffin sections were cut (5 µm) and transferred to glass slides (2 sections per glass slide). After deparaffination, the sections were washed with PBS containing 0.2% (*w*/*v*) IgG-free BSA (Carl Roth, Karlsruhe, Germany). After three washing steps, tissue sections were treated with trypsin (0.06% *w*/*v*) (Germed, Dresden, Germany) for 10 min at room temperature to unmask polySia chains. After three additional washing steps, the tissue sections were blocked with PBS containing 2% BSA (*w*/*v*) for 1 h at 37 °C. After three more washing steps, one section per glass slide was treated with endoN overnight at 37 °C (67 µg/mL in TBS) to degrade polySia. Only TBS was added to the second section per glass slide. Thereafter, the sections were washed three times. As described for Western blotting, polySia was visualized with mAb 735 (2 µg/mL in PBS containing 0.2% (*w*/*v*) IgG-free BSA, overnight at 4 °C). Thereafter, the sections were washed and incubated for 1 h at room temperature with a secondary antibody (Dako, envision kit+ system-HRP labeled polymer anti-mouse, Jena, Germany), as described earlier [37]. After additional washing steps, a peroxidase chromogen for immunohistochemistry (IHC) SIGMAFAST 3,3′-diaminobenzidine (DAB)-tab (Sigma-Aldrich, St. Louis, MO, USA) was added. The nuclei were counterstained with hematoxylin for 10–15 s. Pictures were taken using a transmitted light microscope (AXIO, Carl Zeiss, Oberkochen, Deutschland).

### 2.6. Statistical Analysis

Neu5Ac-Data sets were analyzed by Graph Pad Prism 9.5 software using ANOVA and a multiple comparison Tukey test. Significant differences were as follows: * *p* ≤ 0.05; ** *p* ≤ 0.01; *** *p* ≤ 0.001; ****p ≤ 0.0001.

## 3. Results

### 3.1. Neu5Ac Concentrations Rapidly Decrease during Early Lactation

In mammals, polySia chains seem to consist only of Neu5Ac residues [13,14,38]. Earlier studies described that in cows, the Neu5Ac concentration and, thus, the basic building block of polySia quickly decrease during the first three days of lactation [1,39]. For this reason, we collected milk samples from German Holstein cows on the day of calving and on each of the following two days. Additionally, samples of mature milk were included, which were collected before and after calving. To verify whether the described decrease in Neu5Ac during the first days of lactation also took place in the selected German Holstein cows, the Neu5Ac concentrations in these samples were determined. To this end, the Neu5Ac residues were released under acidic conditions and fluorescently labeled with DMB. Subsequently, the resulting DMB-Neu5Ac was analyzed using an HPLC-system equipped with a C-18 column.

**Figure 1:**
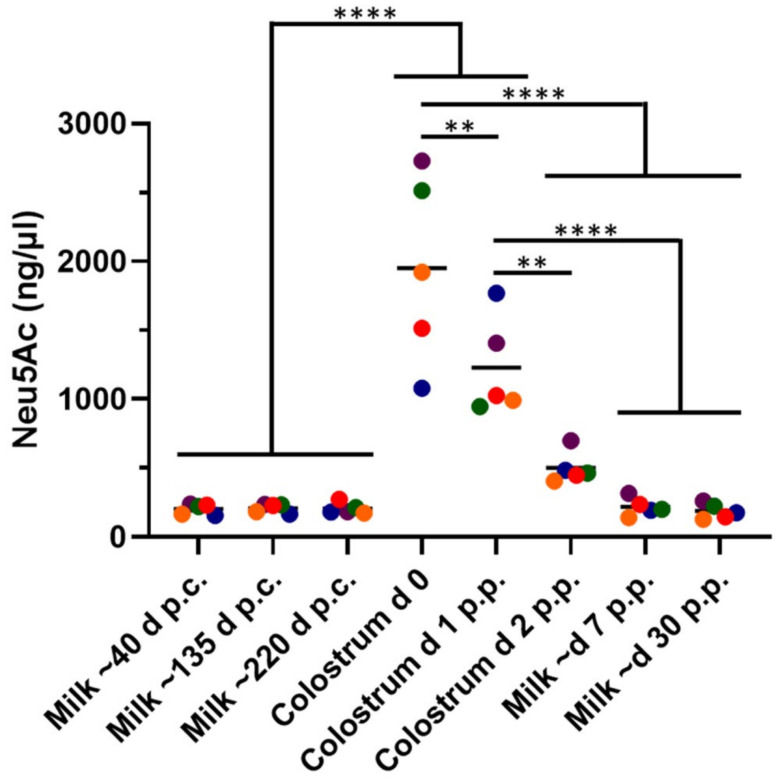
The Neu5Ac concentration decreased during early lactation. The amount of Neu5Ac was determined by RP chromatography after DMB-labeling. Milk samples of five animals were collected at the given time points. The Neu5Ac concentrations of each animal are displayed in the diagram, in addition to the mean values. ANOVA and multiple comparison Tukey test were applied. Significant differences were as follows: ** *p* ≤ 0.01; **** *p* ≤ 0.0001. p.c. = post conception, p.p. = postpartum, d = day.

On the day of calving, the values for Neu5Ac varied between 1079 and 2728 ng/µL (Figure 1). The concentration of Neu5Ac decreased, in accordance with previous studies, during the first days of lactation [1,39]. In contrast to colostrum, in mature milk, the amounts of Neu5Ac were more constant, and values of approximately 200 ng/µL are common. Thus, the concentration of Neu5Ac decreased by up to 13 times during early lactation in German Holstein cows.

### 3.2. The Amount of PolySia Decreases in Line with the Neu5Ac Concentrations

The same milk samples were used to detect Neu5Ac polymers. For this purpose, polySia was isolated with an enzymatically inactive form of endoN. This was possible because endoN contains, in addition to its active site, a binding domain for Neu5Ac polymers [40,41]. The inactive endoN was covalently coupled to magnetic beads and was used for the enrichment of the carbohydrate polymer. The resulting eluates were analyzed by Western blotting, using a mAb against polySia. For negative control, polySia was degraded with active endoN to abolish the binding of the primary antibody.

**Figure 2:**
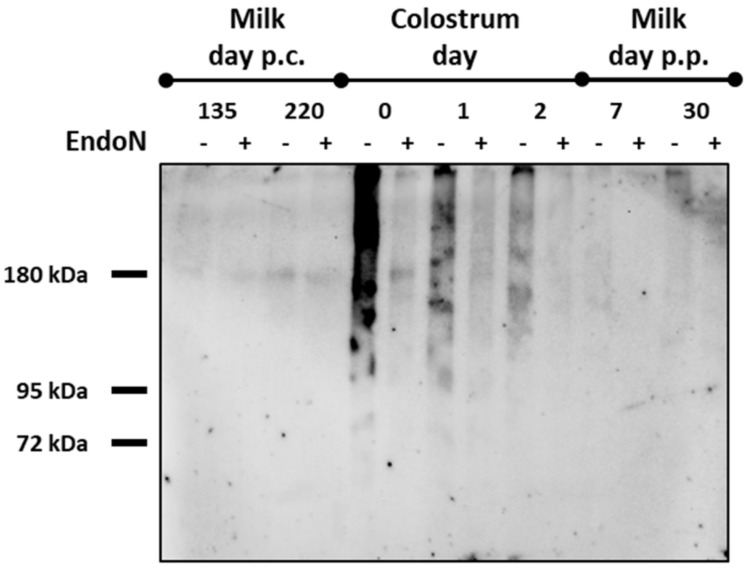
The polySia levels decrease during early lactation in the milk of dairy cows. PolySia was isolated from milk via magnetic beads, which were coated with inactive endoN. The eluates were used for Western blotting against polySia. Specific binding of mAb 735 was confirmed by pretreatment with endoN. PolySia contents in milk were analyzed at different time points of lactation. p.c. = post conception, p.p. = postpartum.

On the day of calving, a strong polySia signal occurred, which almost disappeared, when the samples were treated with endoN (Figure 2). Thus, an unambiguous detection of polySia was possible. The obtained diffuse signal over a wide molecular weight range was typical for polySia, because a heterogeneous chain length distribution usually occurs in vivo [17,36]. In line with the Neu5Ac concentration, the polySia signal decreased during the first three days, and in mature milk, the differences between endoN-treated and untreated samples were difficult to detect.

### 3.3. Both Polysialyltransferases Are Expressed in Udder Tissue

**Figure 3:**
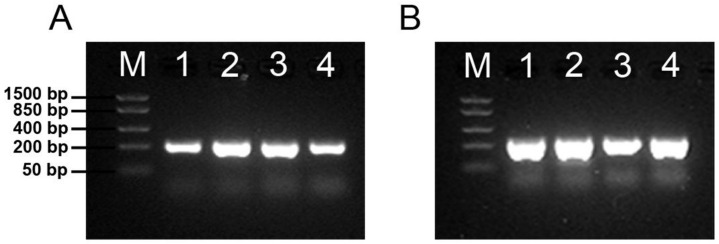
mRNA expression of bovine polysialyltransferases. Agarose gel electrophoresis of RT-PCR amplicons of *ST8SIA2* (**A**) and *ST8SIA4* (**B**) from total RNA of bovine udder tissue (1), brain (2), lung (3), and cultured primary bovine MECs (4). Lane M; FastRuler™ Low Range DNA Ladder (Thermo Scientific^TM^).

Two enzymes, the polysialyltransferases ST8SiaII and ST8SiaIV, can synthesize polySia in mammals [13]. To determine the expressed polysialyltransferases, the transcription of *ST8SIA2* and *ST8SIA4* was investigated in udder tissue. As positive controls, mRNA samples from bovine brain and lung tissue were analyzed in parallel. These two tissues are known to express both polysialyltransferases [14]. As shown in Figure 3, signals for both polysialyltransferases were detectable in these control tissues. In udder tissue, *ST8SIA2* and *ST8SIA4* were also expressed.

Since the analyzed udder tissue contains different cell types, mRNA from MECs was additionally used. The obtained results demonstrated that cultivated epithelial cells, which were enriched from udder tissue, also expressed *ST8SIA2* and *ST8SIA4*. This result suggests that epithelial cells are a source of polySia in milk and that both polysialyltransferases are involved in biosynthesis.

### 3.4. PolySia Is Located in Epithelial Cells in Udder Tissue

To localize polySia in the udder, formalin-fixed tissue was used, which was embedded in paraffin. The tissue sections were deparaffinized and rehydrated. Subsequently, polySia was visualized by immunohistochemistry with the same mAb as was already used for Western blotting. For the negative control, endoN-treated tissue was analyzed in parallel on the same slide. In udder tissue, epithelial cells showed immunostaining (Supplemental Figure S1). However, comparable signals were also observed in endoN-treated samples. Thus, endoN failed to degrade polySia or nonspecific staining occurred.

**Figure 4:**
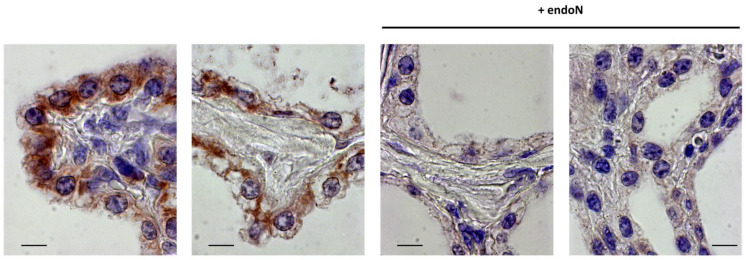
Visualization of polySia in udder tissue. For the immunohistochemical localization of polySia in udder, tissue sections were pretreated with trypsin and stained with the mAb 735 against polySia. For the negative control, the tissue samples were additionally incubated with endoN to degrade polySia. The sections were counterstained with hematoxylin. Scale bar: 10 µm.

Since polySia interacts with lactoferrin in milk, formalin-fixed or, rather, cross-linked lactoferrin may prevent the binding of endoN to polySia and, thus, the degradation of polySia. To prove this hypothesis, tissue sections were pretreated with trypsin. As shown in Figure 4, the application of endoN to trypsinized tissue showed no immunostaining against polySia. In contrast, this pretreatment of the tissue with trypsin does not affect the polySia staining. This result suggests that the application of trypsin leads to an unmasking of polySia for endoN by a proteolytic degradation of cross-linked interaction partners. In summary, the obtained results revealed that polySia is produced in epithelial cells in udder tissue.

### 3.5. Analysis of PolySia in Milk of Different Farm Animals and Human Milk

**Figure 5:**
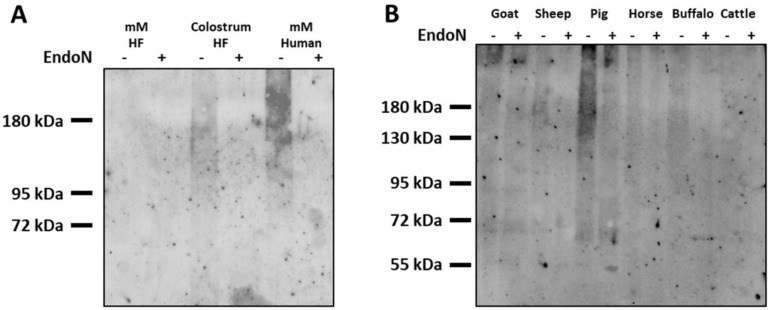
PolySia levels in milk of different farm animals and humans. PolySia was isolated by affinity precipitation and analyzed by Western blotting, as described in Figure 2. (**A**) PolySia from human milk samples was visualized in parallel to eluates from bovine (HF) colostrum and mature milk. (**B**) The mature milk of different farm animals was used for the isolation and subsequent detection of polySia by Western blotting. Pretreatment with endoN served as a negative control. mM = mature milk.

In contrast to bovine milk, an increase in the polySia-levels was observed in humans, when colostrum and mature milk samples were analyzed [16]. To obtain an impression of how the polySia amount differs between human and bovine milk, we analyzed human mature milk in parallel to bovine colostrum and mature milk. The signal against polySia of human milk samples was even stronger than the immune signal of bovine colostrum (Figure 5A). Thus, clear differences between human and bovine milk were observed with regard to the polySia signal intensity of mature milk.

Moreover, the polySia content in mature milk of further farm animals was analyzed by Western blotting. The strongest polySia signals were detectable in porcine milk (Figure 5B). In milk samples of horses and ruminants, only minor or no differences between endoN-treated and untreated samples were observed. Thus, the polySia levels are close to or under the limit of detection.

Taken together, dairy cows as well as other ruminants seem to secrete lower amounts of polySia than humans into mature milk and only in porcine milk was a strong polySia signal also detectable. However, detailed sample collections, as used for the analysis of milk from Holstein–Friesian dairy cows, are needed to make accurate statements about the sialylation status in the additionally analyzed milk samples of other mammals.

## 4. Discussion

Milk is not only an important source of nutrients but also contains numerous bioactive compounds, such as growth factors, immunoglobulins (Igs), lactoferrin, and oligosaccharides. These biofunctional molecules are essential for the optimal vitality of offspring. For instance, in the case of Igs, it is well known that a transfer of colostral Igs to the bloodstream of calves is an essential process [42,43]. Remarkably, the amounts of most bioactive components drop very quickly during the first three days of lactation in dairy cows [43,44]. The same applies for Neu5Ac, which represents an important biological regulator as a part of oligo- and polysaccharides [2,45,46,47,48].

In the analyzed milk samples of German Holstein cows, we also observed a rapid decrease in the Neu5Ac concentration. Moreover, it is striking that there are large differences between the individual animals in the daily measured Neu5Ac concentrations during the first three days of lactation. On the day of calving, high and very heterogeneous Neu5Ac concentrations were observed (between 1079 and 2728 ng/µL), whereas in mature milk, values between 129 and 317 ng/µL were common. Already during the first day of lactation, these significant changes occurred (day 1 p.c. 946–1770 ng/µL; day 2 p.c. 407–698 ng/µL), which is in line with other bioactive biomolecules, such as Igs [43,44].

The rapid decrease and the heterogeneous concentration of Neu5Ac might have a critical impact on the immunological situation in the udder, since sialic acids are very important self-associated molecular patterns (SAMPs) [45]. Sialylated structures are recognized by sialic-acid-binding immunoglobulin-like lectins (siglecs) of immune cells, which can prevent extreme activation of the immune system [47,49,50]. For instance, it was shown that in murine and human blood systems, sialic acids are important to prevent an exaggerated reaction of neutrophils during inflammation [51]. Comparable effects of sialic acids on cervical mucins were described in cattle, where the formation of neutrophil extracellular traps (NETs) is inhibited by a sialic acid-dependent mechanism [52]. NETs are meshworks of DNA and several antimicrobial components, such as histones, neutrophil elastase, and lactoferrin, and are important for enclosing pathogens in an antimicrobial environment. However, excessive NET formation can lead to pathological consequences, such as vascular occlusion and sepsis [53,54,55]. Remarkably, it was recently shown that the formation of blood NETs also increases the risk for mastitis during the transition period of dairy cows [56]. In sheep, NETs are also formed in mammary glands during mastitis [57], and these molecular meshes might increase the risk of a blockage of milk ducts. Since high levels of NETs are also toxic to bovine mammary epithelial cells [58], sialylated components in milk might have a positive effect in preventing exaggerated NET formation during the transition phase and, thus, additional mammary gland damage during mastitis.

Our results indicate that, in line with the Neu5Ac levels in milk, polySia levels also decrease massively during the first three days of lactation. Both polysialyltransferases, ST8SiaII and ST8SiaIV, seem to be involved in the production of polySia in the udder, and MECs are one source of the detected polySia in milk. Whether MECs are solely responsible for the high polySia concentrations in colostrum remains unclear. Due to the open blood–milk barrier during the first days of lactation, large molecules also passively enter the colostrum in paracellular form, while others are actively enriched in the colostrum transcellularly by MECs [59].

Intriguingly, in addition to monosialyl residues, sialic acid polymers also influence the activation of immune cells and the formation, as well as the activity of, NETs [50]. Thus, these secreted polymers might also influence immunological processes in the udder and/or calves. For instance, in NETs, histones have an important function as antimicrobial components [60]. However, these extracellular histones seem to be the major reason for epithelial cell death after exaggerated NET-release [53]. Remarkably, polySia binds histones and efficiently inactivates their cytotoxicity against endogenous cells [36,61,62]. In contrast, the main histones in NET, histone H2A and H2B, retain their antimicrobial activity in the presence of polySia [31]. Furthermore, polySia supports lactoferrin in its function of preventing the release of NET [17,63]. Moreover, polySia can act as an inhibitor during LPS stimulation, when it is recognized by the siglecs of immune cells [64,65,66]. Thus, polySia might be involved in several immunological mechanisms of the udder and the offspring.

In addition to the outlined impact on the mechanisms of immune cells, soluble sialylated oligo- and polysaccharides show anti-adhesive properties against pathogenic microorganisms and viruses [7,8,67]. This is possible because pathogens frequently use sialylated structures of the epithelial glycocalyx to adhere and, thus, initiate an invasion and colonization. Influenza viruses are probably the best known example [2]. Soluble sialylated structures block the sialic receptors of pathogens to prevent binding to the glycocalyx and, thus, to the cell surface. This might also be important for the health of udders and calves during the transition phase [7,8,67].

However, the low amounts of Neu5Ac and polySia in the mature milk of dairy cows seem not to be unusual in comparison with the amounts in other ruminants and horses. Only in pigs and humans were higher amounts detected in mature milk. Nevertheless, it is not clear if, in other ruminants and horses, such a rapid decrease of sialylated structures takes place and if such striking differences in the Neu5Ac levels occur between the individual animals. This should be investigated in more detail in future studies using detailed sample collections, as were used for the analysis of milk from Holstein–Friesian dairy cows.

## 5. Conclusions

Since bovine colostrum shows several therapeutic benefits [43], our results strongly suggest that the sialic acid and, probably, the polySia levels might be interesting markers for quality control, when colostrum is used in human and veterinary health. One may assume that the time of milk collection alone is not indicative of the actual biological activity of bovine milk, since remarkable individual differences for Neu5Ac were detected in German Holstein cows. Thus, the outlined sialic-acid-dependent benefits may also vary in the same way.

## Data Availability

Not applicable.

## Supplementary Materials

The following supporting information can be downloaded at www.mdpi.com/article/10.3390/biology12010005/s1: Figure S1: Visualization of polySia in udder tissue.
