# Supplementary material for: Milk Polysialic Acid Levels Rapidly Decrease in Line with the N-Acetylneuraminic Acid Concentrations during Early Lactation in Dairy Cows
Source: Biology (Basel). 2022 Dec 20;12(1):5. doi: 10.3390/biology12010005 (PMC9854834; doi:10.3390/biology12010005)
Supplement: Supplementary file 1 [file biology-12-00005-s001.zip › biology-2072002-supplementary.pdf]

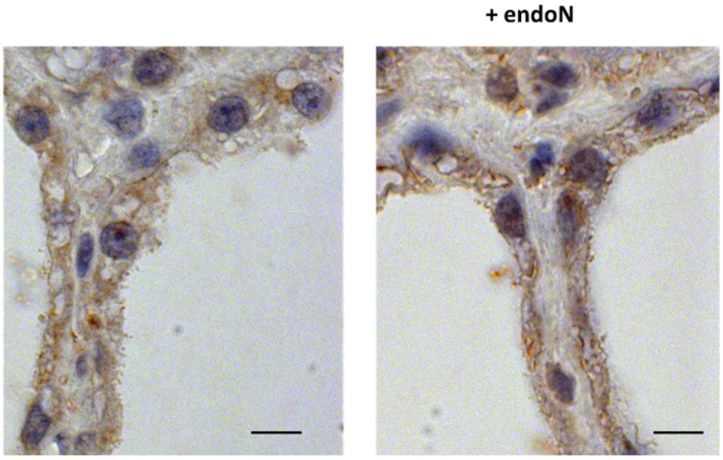

Supplemental Figure S1: Visualization of polySia in udder tissue. For the immunohistochemical localization of polySia in udder, tissue sections were stained with the mAb 735 against polySia. For the negative control, the tissue samples were pretreated with endoN to degrade polySia. The sections were counterstained with hematoxylin. Scale bar: 10 µm.
